# Application of Bayes' Theorem in Valuating Depression Tests Performance

**DOI:** 10.3389/fpsyg.2018.01240

**Published:** 2018-07-23

**Authors:** Marco Tommasi, Grazia Ferrara, Aristide Saggino

**Affiliations:** Department of Psychological, Health and Territorial Sciences, Università degli Studi G. d'Annunzio Chieti e Pescara, Chieti, Italy

**Keywords:** diagnostic accuracy, Bayes' theorem, depression, clinical psychology, sensitivity and specificity

## Abstract

The validity of clinical diagnoses is a fundamental topic in clinical psychology, because now there are some political administrations, as the IOM or the U.K. government, which are focusing on best evidence-based practice in clinical psychology. The most problematic issue in clinical psychology is to avoid wrong diagnoses which can have negative consequences on individual life and on the utility of clinical treatments. In the case of diagnoses based on self-report tests, the diagnostic decision about individual health is based on the comparison between its score and the cutoff, according to the frequentist approach to probability. However, the frequentist approach underestimates the possible risks of incorrect diagnoses based on cutoffs only. The Bayesian approach is a valid alternative to make diagnoses on the basis of the scores from psychological tests. The Bayes' theorem estimates the posterior probability of the presence of a pathology on the basis of the knowledge about the diffusion of this pathology (prior probability) and of the knowledge of sensitivity and specificity values of the test. With all this information, it is possible to estimate the diagnostic accuracy of some self-report tests used for assessing depression. We analyzed the diagnostic accuracy of the most used psychological tests of depression (Zung's Self-Rating Depression Scale, Hamilton Rating Scale for Depression, Center for Epidemiological Studies for Depression and the Beck Depression Inventory), together with a new scale (Teate Depression Inventory) developed with the IRT procedure, by analyzing the published works in which data about sensitivity and specificity of these scales are reported. Except the TDI, none of these scales can reach a satisfactory level of diagnostic accuracy, probably for the absence of an optimal procedure to select test items and subjects with clearly defined pathological symptoms which could allow the reduction of false positives in test scoring.

## Introduction

In these last years the problem of validity of psychological diagnoses has become an important topic in psychological research (Witteman et al., 2007). The principal focus is the development of psychological instruments, in particular self-report measures, which have resulted to be more and more efficient in detecting individuals suffering from psychological syndromes, as depression, obsessive-compulsive disorders, anxiety, etc. This is, really, an old topic in clinical psychology (Meehl and Rosen, 1955), but in the last years the necessity of a more precision in diagnostic accuracy also in psychology has become ever more relevant (Witteman et al., 2007; Westbury, 2010; Colquhoun, 2014).

For example, the relatively recent focus on best evidence-based practice in clinical psychology has been substantially influenced by the Institute of Medicine (IOM) reports on the research and policy to deal with problem of mental health and substance use disorders. These works of the National Academies played an important role in the public health policies of the Patient Protection and Affordable Care Act (ACA or Obamacare) as well as of the Mental Health Parity and Addiction Equity Act. The ACA, in addition to the expansion of medical therapies coverage, reformed the procedure by which medical care is delivered to people, with a strong emphasis on accountability and performance measurement of therapists, whereas the Mental Health Parity and Addiction Equity Act attempted to ensure that individuals with mental health and substance use problems could have access to behavioral health care services (Barlow, 2015).

On the other hand, in the United Kingdom a program was developed, defined as the English Improving Access to Psychological Therapies (IAPT), which was intended to provide evidence-based psychological therapies to a general population (Gyani et al., 2013). The rationale was that if people suffering of mental diseases as, for example, anxiety and depression, could obtain valid psychological assistance, then there was not only a benefit for the single individual, but also for the entire society, even from the economic standpoint. However only a quarter of people suffering of mental disease are actually in treatment in Europe and in the U.S.A. (Layard, 2013).

A way to allow people suffering of mental disease to obtain a valid psychological treatment is improving the accuracy of psychological diagnosing in clinical settings. The efficacy of psychological treatments is strongly connected with the accuracy of psychological diagnosing (Barlow et al., 2013; Gyani et al., 2013).

In medical practice many attempts were made to improve diagnostic accuracy (Begg, 1987; Hui and Zhou, 1998; Zhou, 1998), and one of the most promising procedure was the Bayes' theorem (Van Stralen et al., 2009). The Bayes' theorem (also defined Bayes' law or Bayes' rule) is, really, an equation which estimates the probability of an event on the basis of the prior knowledge of conditions that are related to the event (Glickman and Van Dyk, 2007; Viertl, 2012). The Bayesian approach to probability, or bayesianism, is different from the frequentist approach, or frequentism, because it takes into account the level of belief of a person when she/he has to estimate the occurrence probability of an event. This approach is also defined “subjective probability” (Fienberg, 2006). Frequentism, on the other side, take into account only the probability that the event occurrence is due only to random factors. Therefore, the probability on an event can be determined only after its occurrence. In this case, frequentism is related to the likelihood function of the events. The frequentism was the preferred approach to estimate the probability of an event for many years (Fienberg, 2006). It is with the works of some authors (Wald, 1950; Savage, 1951, 1961) that from 50s the Bayes' theorem started to be accepted in the field of statistics and mathematical applications of probability.

The application of frequentism needs the existence of a frequency (density) distribution of a specific characteristic in population. To create the density distribution is necessary a long series of successive trials or observations to count the occurrence of successes or failures. For example, to estimate the probability of head or tail after tossing a coin, we can toss for 5 thousand times, and count how many times we have obtained heads. If the result of an event is due only to random factors, as in the case of tossing a coin, the frequency of head occurrences is equivalent to that of tail occurrences. Therefore both head and cross have the probability of 0.5 to occur. Results of random events have the same probability to occur. In the case of tossing a dice, each face of the dice has 1/6 probability to occur. However, there are some events for which is not possible to estimate the density distribution. For example, Laplace estimated the error probability of calculating the mass of Saturn, but the mass of the planet is not a random variable. Therefore, there are empirical situations in which the classic theory of probability cannot be used (Viertl, 2012).

Also in the field of psychology, some psychological characteristics have a density distribution (e.g., intelligence, personality traits) and thanks to these density distributions we can establish if an individual is normal or pathological. The problem of density distribution of psychological syndromes is relevant especially in diagnostic accuracy in clinical psychology, wherein it is necessary to evaluate if an individual is normal or pathological (Westbury, 2010).

One of the major limits in clinical psychology is the absence of *gold standard* tests or, in other words, tests which have a 100% of sensitivity and 100% of specificity (Black et al., 1999; Black and Craig, 2002). In absence of a perfect test, therefore, multiple imperfects tests are used in order to gain an improved estimate. In general, the results of these test are correlated, given a subject's disease status (Black et al., 1999; Black and Craig, 2002). However, because tests are imperfect the correct interpretation of their results is always at risk (Lesaffre et al., 2007). It is necessary to add information not present in the data collected with the test and this further information is our knowledge about the parameter of interest (e.g., the probability than a human being is affected by a mental disorder) before performing the test. This previous knowledge constitutes the prior probability of the parameter and the bayesian analysis allows the combination of this prior probability with the collected data to yield an estimate (Black et al., 1999; Black and Craig, 2002; Lesaffre et al., 2007). In addition, Bayes's theorem can be applied in different kinds of analyses, e.g., to analyze individual scores obtained in successive performances to estimate if test failure can be reduced or not (Sheppard and Kaufman, 2005), to reduce failure in radiological examinations (Santosh and Antani, 2018) or to improve analysis of documents with incomplete data (Philippot et al., 2015).

When psychological tests are used to estimate normality, psychometricians or clinical psychologists define specific test scores, the cutoffs, which have the function of delimiting the area of normality. Usually personality traits have a specific distribution of probability which is used to define the cutoff for judging an individual normal or pathological. When an individual obtains a test score which overcomes or it is outside the boundaries defined by cutoffs, this individual is considered not normal or not belonging to normal population. Usually, the most used cutoffs to define the boundaries of normality are 1.96 or 1.64, if raw scores are transformed into *z* scores. In personality tests the most used cutoff value is 65, when raw scores are transformed into standardized T scores and in intelligence tests the most used cutoffs are 70 and 130, when raw scores are transformed into standardized QI scores.

On the basis of test scores, psychologists determine the normality of examinees and these scores, compared to the relative cutoffs, allow psychologists to decide if the individual does not show particular problems or, otherwise, if she/he presents some psychological diseases or impairments. The rationale on which this procedure is based is that extreme scores are very rare and, therefore, the individual score is probably affected by other factors beyond random variance.

Density distributions of tests scores are used to estimate if an individual is normal or clinical. Cutoffs define the portion of area under the density distribution curve in which individual can be considered, with a certain amount of error, normal or pathological. The amount of error is, usually, 5 or 2.5%. These percentages indicate the risk to obtain false positives or to judge an individual as pathological when, actually, he is normal. If the density distribution is composed by scores obtained by a sample of non-clinical or normal subjects, cutoffs divide the area of the probability curve into the area of false positives (FPs) and of true negatives (TNs). If it is possible to collect scores from a sample of clinical or pathological subjects, then cutoffs divide the area of the density distribution into the area of false negatives (FNs) and the area of true positives (TPs). FPs and TPs are those subjects who overcome the cutoffs. The biggest risk in diagnostic settings is to judge a person as pathological while, actually, he is perfectly sane, or to judge a person as normal, when he is severely pathological. These errors can severely worsen individual life. Therefore, it is necessary an accurate estimation of risk while doing diagnoses.

A very good psychological test should have a low percentage of FPs and a high percentage of TPs. Frequentism considers only the risk to commit FPs (Colquhoun, 2014). For example, if for a specific test, we define a cutoff by which there is only 5% of FPs, however this is not the true level of risk probability. We have to estimate also the percentage of TPs, and then to estimate the False Positive Rate (FPR) which is given by the equation FPs/(FPs + TPs). For example, if a psychological test for diagnosing the presence of a depressive syndrome has a 5% of FPs, that is equivalent to 45 subjects on a total of 900 normal subjects, and a 80% of TPs, that is equivalent to 80 subjects on a total of 100 pathological subjects, then the FPR is 45/(45 + 80) = 0.36. In other words, we have about 36% of probability to commit a mistake when we decide that a subject is pathological. This percentage is clearly superior than the usual level of error of 5%. We have to say that psychological tests with a low percentage of FPs (about 5%) are very rare and that many tests have, actually, a greater percentage of FPs. In addition, even a percentage of 80% of TPs is not very common. Therefore, the FPR value could be very high in diagnostic settings.

However, bayesianism poses another important question, which usually is not considered in estimating the risk of errors in diagnostic accuracy. Bayes' theorem is a statement about conditional probabilities that does not allow the exchange of the order of the events. In other words, if A and B are two events, the occurrence probability of the event A, given B, is not the same of the occurrence probability of the event B, given A (Glickman and Van Dyk, 2007; Viertl, 2012). In the case of psychological assessment, if we develop a test to estimate if a person is normal or pathological, the probability that this person is pathological, if she/he resulted positive at the test is not equivalent to the probability to obtain a positive result, if she/he is truly pathological.

By supposing that we are using a psychological test for diagnosing the presence of depression in people, the probability of a person to be actually depressed, if her/his score overcomes the cutoff value is not equivalent to the probability to obtain scores higher than the cutoff, when she/he is effectively depressed. In other words, when we have to do a diagnosis, to estimate the actual risk of a failure, we have to consider the conditional probability of a person to be depressed when she/he overcomes the cutoff and also the conditional probability of a person to overcome the cutoff when she/he is depressed. Let A the probability of a person to be actually depressed and B the probability to overcome the cutoff. According to Bayes' theorem, the conditional probability of a person to be depressed when he overcomes the cutoff (P(A|B)), also called the posterior probability, is:

(1) $$\begin{array}{r} \text{P}\left(\text{A}|\text{B}\right)=\left[\text{P}\left(\text{B}|\text{A}\right)\text{P}\left(\text{A}\right)\right]/\text{P}\left(\text{B}\right) \end{array}$$

wherein P(B|A) is the conditional probability to overcome the cutoff when the person is actually depressed (the probability of TPs), P(A) is the probability to be depressed in the population and P(B) is the probability to obtain a test score that is higher than the cutoff. P(A) is called the prior probability of A, and is related to the percentage of people who are actually depressed in the population. For example, the percentage of depressed people in the U.S.A. population is 6.7% (source: https://www.nimh.nih.gov/health/statistics/major-depression.shtml). Therefore P(A) = 0.067. The probability P(B) is given by the sum of the percentage of FPs and TPs. To estimate P(B) we should know, therefore, the number of FPs and TPs for each specific test used to measure the level of depression. Let us suppose that the percentages of FPs and TPs is 5 and 80% respectively. The conditional probability that a person overcomes the cutoff, if he is actually depressed, is P(B|A) = 0.8, while the probability that a person overcomes the cutoff independently by his pathological state is P(B) = 0.05(1 − 0.067) + 0.8(0.067) = 0.10. The prior probability to be depressed is P(A) = 0.067. The conditional probability that a person is actually depressed if he overcomes the cutoff is: [P(B|A)P(A)]/P(B) = [0.8(0.067)]/0.10 = 0.54.

**Figure 1:**
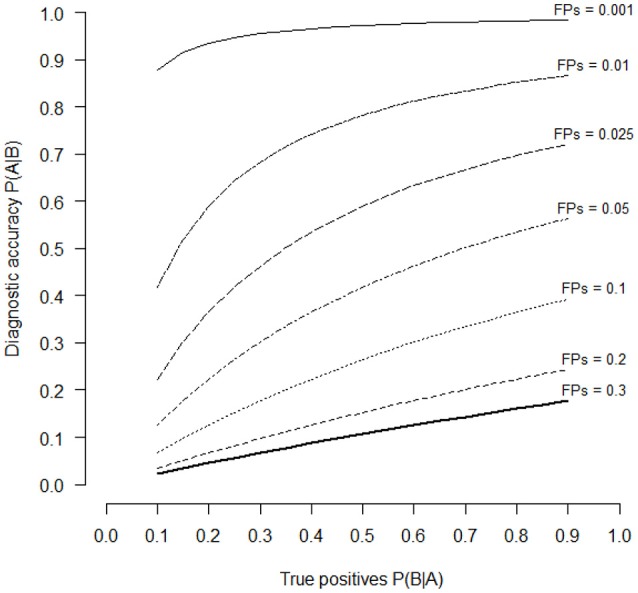
Diagnostic accuracy of an hypothetical psychological test in relation to the different levels of TPs (P(A|B) values of the horizontal axis) and of FPs (series of curves with different line styles). Diagnostic accuracy is estimated with the Bayes' theorem (Equation 1). The selected levels of FPs were 30, 20, 10, 5, 2.5, 1, and 0.1%.

In other words, the probability that the individual is actually depressed is only 54%. Surely the probability is superior than the probability due to pure chance (50%), however it is not a really high value. There are different reasons for this result. The first reason is that the probability of depressed people in the population is (fortunately) low (about 6.7% of the population suffer from a severe depression). The second is that the test has not a very high percentage of TPs (about 80%), even if psychological tests with usually 80% of TPs are considered very good tests. The third reason is that a percentage of 5% of FPs reduces the probability to recognize correctly depressed people. Because we cannot reduce the percentage of depressed people in population and that the percentage of 80% of TPs is usually considered a good percentage, the remaining thing to do is to improve test reliability by reducing the percentage of FPs. For example, if we reduce from 0.05 to 0.025 the proportion of FPs, then P(B) = 0.025(1 − 0.067) + 0.8(0.067) = 0.08. Therefore, [P(B|A)P(A)]/P(B) = [0.8 (0.067)]/0.08 = 0.67. By halving the proportion of FPs we have increased the probability to correctly detect depressed people from 54 to 67%. Figure 1 shows the variation of the conditional probabilities P(A|B) against different values of P(B|A) (the proportions of TPs) in relation to the proportions of FPs for tests which have to recognize depressed people in the population (with a prior probability of 6.7%). The selected FPs values were 0.3, 0.2, 0.1, 0.05, 0.025, 0.01, and 0.001. The proportions of TPs or P(B|A) values represent also the power of the psychological tests or the capacity of the test to correctly recognize pathological persons.

It is possible to see that only when the proportion of FPs is lower than 0.05, psychological tests are able to correctly identify depressed people. By reducing the level of FPs it is possible to increase the probability to make a correct diagnosis of depression. If we set P(A|B) = 0.50, which is the same probability to guess the correct diagnosis by chance, the power of the test P(B|A) is 0.70, 0.34, 0.16, and < 0.10, if the proportions of FPs are 0.05, 0.025, 0.01 and 0.001 respectively. If we set P(A|B) = 0.75, which means to make a correct diagnosis with a percentage greater than pure chance, the necessary power to detect depressed people P(B|A) is 0.41 and < 0.10, for FPs proportions of 0.01 and 0.001 respectively. Therefore, to increase the probability to make a correct diagnosis, psychological tests should have a low proportion of FPs and a relatively high proportion of TPs.

Obviously, the levels of P(A|B) are not only affected by the proportion of FPs and TPs, but also by the proportion of pathological individuals in the population. In the case of psychological depression the proportion of depressed people, always making reference to the U.S.A. population, is affected by gender: 4.8% of males and 8.5% of females are depressed, respectively. Depression is more frequent in young people: 10.9% of people with age between 18 and 25 years are depressed against the value of 6.7% in the general population. This means that psychological tests have higher P(A|B) values in females or young people than in adult males because of the higher occurrence of the pathology in these groups of people.

Independently by the presence of depression among people, it is necessary to analyze if psychological tests are reliable instruments for making accurate diagnoses of depression. Therefore we decided to consider the most used and famous test currently used to make diagnosis of depression: the Beck Depression Inventory, the Hamilton Depression Rating Scale, the Center for Epidemiologic Studies Depression Scale and the Zung Self-Rating Depression Scale. In addition to these scales, we also included a new scale for depression the Teate Depression Inventory.

For each depression scale we collected papers wherein the sensitivity, specificity and cutoff of each scale are reported. Sensitivity is the proportion of TPs, or sensitivity = TP/(TP + FN), while specificity is the proportion of True Negatives (TNs), or specificity = TN/(TN + FP). The proportion of FPs is equivalent to 1 minus the proportion of TNs. Therefore, 1 minus the specificity gives the proportion of FPs, which is the probability to incorrectly recognize depressed people. The proportion of TPs (sensitivity) is the probability P(B|A) and represents the power of the psychological test (probability to recognize correctly depressed people). Therefore, the probability P(B) to obtain a positive result in a test is given by the following formula: P(B) = [sensitivity × P(A)] + [(1 – specificity) × (1 – P(A))], where P(A) is the prior probability of being depressed. For each depression scale, we also collected the Positive Predictive Value (PPV) and the Negative Predictive Value (NPV). PPV is defined as the probability that a person randomly chosen from the population who has tested positive actually has the disease, and NPV is the probability that a person who has tested negative does not have the disease (Hui and Zhou, 1998). PPV is the proportion of patients with positive test result in total of subjects with positive result (PPV = TP/[TP + FP]), while NPV is the proportion of subjects without the disease with a negative test result in total of subjects with negative test results (NPV = TN/[TN + FN]) (Šimundić, 2008). Good tests should have PPV and NPV values close to 1 or 100, if values are in percentage (Šimundić, 2008).

## Materials and methods

We selected the four most used self-report psychological tests used to make diagnostic decision about the presence of symptoms of depressive disorders. The four tests were: the Zung Self-Rating Depression Scale (ZSDS), the Hamilton Rating Scale for Depression (HAMD), the Center for Epidemiological Studies for Depression (CES-D) and the Beck Depression Inventory (BDI). We also included another test for measuring depression recently developed by some of the authors of this paper: the Teate Depression Inventory (TDI).

### Self-report depression scales

#### The beck depression inventory (BDI)

It is a 21 item self-report screening inventory designed to measure the severity of depression (Beck et al., 1961). The BDI considers depression as a unitary dimension which can be applied for diagnoses in different psychiatric and medical contexts. In 1996 some of the items of the test were changed, obtaining, in this way a second version of the depression scale, defined BDI-II (Beck et al., 1996). The inventory uses three cut-off scores to discriminate between “mild,” “moderate,” and “severe” depression which are, respectively, 10, 16, and 20 (Beck et al., 1996).

#### The hamilton rating scale for depression (HAMD)

It is a commonly used self-report instrument to assess depression in research and clinical practice (Hamilton, 1960). For clinical purposes, the HAMD is useful to measure the severity of depression in patients suffering of depression in absence of somatic comorbidity. It is a 21 item scale, even if there is also a shorter version of 17 item which is often used as well (Bagby et al., 2004). The cut-off values of the HAMD to be diagnosed as depressed according to the DSM criteria are in the range 15–20 (Faries et al., 2000).

#### The zung self-rating depression scale (ZSDS)

It is a 20 item self-report scale developed by Zung to measure the severity of depressive symptoms (Zung, 1965). The scale can distinguish between different level of severity in depressive symptoms (Zung, 1973): normal (index scores 25–49); mild to moderate (index scores 50–59); moderate to severe (index scores 60–69); severe (index scores >70). Usually the cutoff value used to discriminate between depressed vs. non-depressed is 50 (Dunstan et al., 2017).

#### The center for epidemiological studies for depression (CES-D)

It is a self-report scale developed to be used in studies of the epidemiology of depressive symptoms in the general population (Radloff, 1977). It was designed to measure current level of depressive symptoms with a particular attention toward the depressed mood. The scale consists of 20 items. The CES-D is partially derived from the BDI but it uses a more concise wording. The standard cutoff score is 16 (Zich et al., 1990).

#### The teate depression inventory (TDI)

The TDI (Balsamo and Saggino, 2013, 2014) is a 21-item self-report scale. It was developed via Rasch logistic analysis of responses, within the framework of Item Response Theory (Rasch, 1960). Growing literature suggests that the TDI has promising psychometric properties in both clinical and nonclinical samples (Innamorati et al., 2013; Balsamo et al., 2015a,b,c; Saggino et al., 2017, 2018; Contardi et al., 2018). Additionally, three cutoff scores were recommended in terms of sensitivity, specificity and classification accuracy for screening of varying levels (minimal, mild, moderate, and severe) of depression severity in a group of patients diagnosed with Major Depressive Disorder (Balsamo and Saggino, 2014).

#### Procedure

We selected papers about the five self-report scales of depression wherein the necessary data for estimating the posterior probability of being really depressed are present. Papers were selected on the basis of precedent reviews about the validity of depressive scales (Bagby et al., 2004; Wang and Gorenstein, 2013; Meader et al., 2014; Martinez-Martin et al., 2016) or by doing a research on Psychinfo or Scholar Google databases. Posterior probability P(A|B) can be calculated if the values of sensitivity and specificity of the scale are available. Therefore we selected papers wherein data about sensitivity and specificity, used as criterions to estimate test validity, were reported. In addition, from the selected papers we reported other kind of information: name of authors, years of publication of their work, the typology of subjects used in the research (normal subjects or patients with psychiatric or medical diseases), number of subjects effectively used for collecting responses on tests of depression and the recommended or used cutoffs to estimate define is the subject is normal or pathological. All these data, including sensitivity and specificity of the depression scale estimated by the authors, are reported in Table 1.

**Table 1:** Studies using the five depression scales by authors, publication year, sample typology, sample size, sensitivity, specificity, FPs percentage, positive predictive value (PPV), negative predictive value (NPV), recommended or used cutoff, P(B) and P(A|B) value.

**Depression scales**	**References**	**Sample typology**	**Sample size**	**Sensitivity**	**Specificity**	**FPs percentage**	**PPV**	**NPV**	**Recommended or used cutoff**	**P(B)**	**P(AIB)**
BDI	Aben et al., 2002	Medical	202	80	61.4		22.2	95.7	10	0.41	0.13
	Arnarson et al., 2008	Psychiatric	Normal = 1454; Clinical = 248	82	75		n.r.	n.r.	20/21	0.29	0.19
	Arnau et al., 2001	Medical	333	94	92		54	99	18	0.14	0.46
	Berg et al., 2009	Medical	100	80	76		n.r.	n.r.	10	0.28	0.19
	Bunevicius et al., 2012	Medical	522	89	74		29	98	14	0.30	0.20
	Carney et al., 2009	Medical	140	81	79		n.r.	n.r.	17	0.25	0.22
	Cho and Kim, 1998	Psychiatric	164	90	n.r.	24	n.r.	n.r.	n.r.	0.28	0.21
	Dolle et al., 2012	Psychiatric	141	100	77		n.r.	n.r.	23	0.28	0.24
	Dozois et al., 1998	Normal	1022	81	92		n.r.	n.r.	13	0.13	0.42
	Dutton et al., 2004	Medical	220	87.7	83.9		69.5	94.2	14	0.21	0.28
	Frasure-Smith and Lespérance, 2008	Medical	804	91.2	77.5		n.r.	n.r.	14	0.27	0.23
	Gorenstein et al., 2011	Normal	n.r.	70	87		84.3	77	10	0.17	0.28
	Jones et al., 2005	Medical	174	95.7	78.3		42	99	11	0.27	0.24
	Kang et al., 2013	Medical	423	92	84		n.r.	n.r.	11	0.21	0.29
	Kapci et al., 2008	Psychiatric	Normal = 362; Clinical = 151	77	76		n.r.	n.r.	19	0.28	0.19
	Krefetz et al., 2002	Psychiatric	100	74	70		n.r.	n.r.	24	0.33	0.15
	Kumar et al., 2002	Psychiatric	100	85	83		85	83	21	0.22	0.26
	Leentjens et al., 2000b	Medical	53	67	88		62	90	13/14	0.16	0.29
	Lincoln et al., 2003	Medical	143	91	56		n.r.	n.r.	16	0.47	0.13
	Low and Hubley, 2007	Medical	119	83	88		21	100	14	0.17	0.33
	Lykouras et al., 1998	Medical	150	85.7	86.7		40	97.5	29	0.18	0.32
	Osman et al., 2008	Psychiatric	Normal = 414; Clinical = 167	86.8	56.8		n.r.	n.r.	10	0.46	0.13
	Pérez-Stable et al., 1990	Psychiatric	Normal = 195; Clinical = 70	91	n.r.	58	n.r.	n.r.	10	0.60	0.10
	Perry and Gilbody, 2009	Normal	1166	65.9	67.9		n.r.	n.r.	21	0.34	0.13
	Pohjasvaara et al., 2001	Medical	390	73	67		n.r.	n.r.	10	0.36	0.14
	Rampling et al., 2012	Medical	266	93.8	78.9		49.5	98	15/16	0.26	0.24
	^†^Scogin et al., 1988	Psychiatric	Normal = 57; Clinical = 61	97	77		n.r.	n.r.	5	0.28	0.23
	Seignourel et al., 2008	Psychiatric	582	81	61		45	91	21	0.42	0.13
	Shean and Baldwin, 2008	Normal	395	73.3	84.4		47.8	94.2	10	0.19	0.25
	Sprinkle et al., 2002	Normal	137	90	n.r.	26	n.r.	n.r.	14	0.30	0.20
	Strik et al., 2001	Medical	199	83.8	71.7		33.3	97.9	8	0.32	0.18
	Turner et al., 2012	Medical	72	92	71		n.r.	n.r.	11	0.33	0.19
	Uslu et al., 2008	Psychiatric	Normal = 503; Clinical = 166	77.4	76.8		63.4	84.5	20	0.27	0.19
	Warmenhoven et al., 2012	Medical	61	90	69		n.r.	n.r.	16	0.35	0.17
	Williams et al., 2012	Medical	269	95	60		62	94	7	0.44	0.15
	Zich et al., 1990	Medical	31	100	89		n.r.	n.r.	16	0.17	0.39
HAMD	*Aben et al., 2002	Medical	202	78.7	74.6		36.8	94.7	12	0.29	0.18
	Agrell and Dehlin, 1989	Medical	40	71	87		60	80	10	0.17	0.28
	Berg et al., 2009	Medical	100	80	93		n.r.	n.r.	10	0.12	0.45
	Cho and Kim, 1998	Psychiatric	164	98	n.r.	6	n.r.	n.r.	n.r.	0.12	0.54
	Kang et al., 2013	Medical	423	89	84		n.r.	n.r.	12	0.21	0.29
	*Leentjens et al., 2000a	Medical	63	88	89		74	96	13/14	0.16	0.36
	*Leung et al., 1999	Psychiatric	93	79	80		77	82	15/16	0.24	0.22
	*Mottram et al., 2000	Psychiatric	433	87.5	99.1		99.1	97.1	16	0.07	0.87
	*Naarding et al., 2002	Medical	403	100^a^; 86^b^; 80^c^	93^a^; 84^b^; 92^c^		88^a^; 61^b^; 76^c^	100^a^; 95^b^; 94^c^	5/6^a^; 9/10^b^; 12/13^c^	0.16	0.38
	Quaranta et al., 2008	Medical	143	84.9	84.1		89.8	77.3	11	0.21	0.28
	Roger and Johnson-Greene, 2009	Medical	67	65	56		28	47	2	0.45	0.10
	*Strik et al., 2001	Medical	206	76.3	86		58.8	98.2	12	0.18	0.28
	*Thompson et al., 1998	Normal	703	96	98		n.r.	n.r.	n.r.	0.08	0.78
	*Williams et al., 2012	Medical	269	77	76		69	83	7	0.28	0.19
ZSDS	Agrell and Dehlin, 1989	Medical	40	76	96		93	84	45	0.09	0.58
	Dunstan et al., 2017	Psychiatric	Normal = 289; Clinical = 87	93	69		n.r.	n.r.	50	0.35	0.18
	Fountoulakis et al., 2001	Psychiatric	normal = 120; clinical = 40	90	92.5		n.r.	n.r.	44/45	0.13	0.46
	Gabrys and Peters, 1985	Psychiatric	Normal = 218; Clinical = 369	92	n.r.	23	n.r.	n.r.	40/50	0.28	0.22
	Greenough and Fraser, 1991	Medical	274	92	73		n.r.	n.r.	56	0.31	0.20
	Magruder-Habib et al., 1989	Psychiatric	Normal = 60; Clinical = 112	70	94		n.r.	n.r.	50	0.10	0.46
	Okimoto et al., 1982	Medical	55	76	82		n.r.	n.r.	60	0.22	0.23
	Passik et al., 2001	Medical	60	86.1	66.7		n.r.	n.r.	48	0.37	0.16
	Spitzer et al., 1994	Medical	337	86	74		n.r.	n.r.	50	0.30	0.19
	Zung and Green, 1973	Psychiatric	n.r.	88	n.r.	44	n.r.	n.r.	40	0.47	0.13
CES-D	Agrell and Dehlin, 1989	Medical	39	56	91		82	75	20	0.12	0.31
	**Cheng and Chan, 2005	Psychiatric	398	76 (75)	55 (51)		57(55)	74(72)	12/13 (22/23)	0.49	0.10
	Cho and Kim, 1998	Psychiatric	164	91.3	78.8		62.7	95.9	25	0.26	0.24
	Fechner-Bates et al., 1994	Medical	425	79.5	71.1		n.r.	n.r.	16	0.32	0.16
	Haringsma et al., 2004	Psychiatric	318	83.7^d^; 85^e^	59.8^d^; 64.3^e^		77^d^; 63.1^e^	n.r.	22^d^; 25^e^	0.47	0.12
	Hendrie et al., 1995	Psychiatric	125	82	88		n.r.	n.r.	16	0.17	0.33
	***Irwin et al., 1999	Psychiatric	83^f^; 68^g^	97^f^; 100^g^	84^f^; 92^g^		85^f^; 38^g^	n.r.	4	0.19	0.34
	Jones et al., 2005	Medical	174	95.7	78.9		42.3	33.1	14	0.26	0.25
	Kirmayer et al., 1993	Medical	685	83.3	n.r.	14.4	n.r.	n.r.	16	0.19	0.29
	Parikh et al., 1989	Medical	180	90	86		80	n.r.	16	0.19	0.32
	Pérez-Stable et al., 1990	Medical	Normal = 195; Clinical = 70	83	n.r.	45	n.r.	n.r.	16	0.48	0.12
	Roger and Johnson-Greene, 2009	Medical	67	66	68		34	35	15	0.34	0.13
	Schein and Koenig, 1997	Medical	76	73.1	84		70.4	85.7	16	0.20	0.25
	Schulberg et al., 1985	Psychiatric	1554^h^; 869^i^	88.9^h^; 89.2^i^	70.4^h^; 37.9^i^		23.3^h^; 98.4^i^	35.3^h^; 90.2^i^	27	0.48	0.12
	Shean and Baldwin, 2008	Normal	395	86.7	76.6		41.9	96.7	16	0.28	0.21
	Shinar et al., 1986	Medical	27	73	n.r.	0	84	n.r.	16	0.05	1.00
	Weissman et al., 1977	Psychiatric	235^j^; 60^k^; 61^l^; 50^m^	99^j^; 74^k^; 94^l^; 93^m^	56^j^; 59^k^; 84^l^; 86^m^		n.r.	n.r.	16	0.33	0.18
	Williams et al., 2012	Medical	269	72	70		62	79	12	0.33	0.15
	Zich et al., 1990	Medical	34	100	81		n.r.	n.r.	27	0.24	0.27
TDI	Balsamo and Saggino, 2013	Psychiatric	125	82	98		98	84	36	0.10	0.58

† *Authors used a 13 items version of the BDI*.

* *Authors used the 17 items version of the HAMD*.

** *Authors used two CES-D versions: one with 10 and the other with 20 items. Values relative to the 20 item version are in parentheses*.

*** *Authors used a 10 items version of the CES-D with binary items*.

a *Stroke patients*.

b *Patients with Alzheimer's disease*.

c *Patients with Parkinson's disease*.

d *Patients with major depressive disorder*.

e *Patients with clinically relevant depression*.

f *Psychiatric patients*.

g *community-dwelling elders*.

h *Primary care patients*.

i *Psychiatric patients*.

j *Acute and recovered depressives*.

k *Drug addicts*.

l *Alcoholics*.

m *Schizophrenics*.

*n.r. not reported datum*.

In some works, the authors did not report values of specificity. In these case we used the percentage or proportion of false positives, when available. Other works did not reported the cutoffs, even if sensitivity and specificity were described. Nevertheless, we decided to insert these works in our analysis.

Sensitivity and specificity were used to estimate the conditional probability of being depressed if results at the test are positive or, in other word, the percentage of correct diagnoses (P(A|B)). For the prior distribution of depression P(A) we decided to use the percentage of people suffering of major depressive episode among U.S.A. adult population of 2016 (source: https://www.nimh.nih.gov/health/statistics/major-depression.shtml). According to the SAMSHA survey, about 6.7% of U.S.A. adults suffers of a major depressive episode (4.8% and 8.5% for male and female population, respectively). We used this value to estimate the posterior probability of being depressed according to Bayes' theorem.

## Results

Table 1 shows the name of authors, the year of publication, the typology of subjects used to estimate criterion validity (sensitivity, specificity, PPV and NPV) of the depression scale and the size of each sample. Sample size indicate the number of subjects that effectively answered to depression scales.

Table 1 shows that many studies did not report PPV and NPV values, even if many publications suggest to report these data when analyzing test accuracy (Begg, 1987; Hui and Zhou, 1998; Šimundić, 2008). The mean values of PPV for the BDI, HAMD and CES-D are 47.36, 68.13 and 62.12%, respectively, while the mean values of NPV for the BDI, HAMD and CES-D are 93.31, 87.03 and 70.17%, respectively. For the ZSDS and TDI we have only one value for the PPV, which are 93% and 98%, respectively, and for the NPV which is 84% for both the depression scales. Using the proportion of sensibility and specificity, we estimated the conditional probability P(A|B) for each depression scale. Table 1 shows the values of P(B) and of P(B|A) for each published work divided for each depression scale. Because the TDI is a newly developed scale, there is currently only one study which reports its sensitivity and specificity values.

Table 1 shows also the three typologies of samples used in each work which were: normal, psychiatric or medical sample. The normal sample includes only subjects without particular psychiatric or psychological syndromes; the psychiatric sample includes subjects who received diagnoses of psychiatric or psychological syndromes (e.g., diagnosis of Major Depressive Disorder); the medical sample includes subjects affected by medical diseases (e.g., patients with Parkinson's disease, stroke patients or primary care patients). The psychiatric sample could indicates also a mixed sample or, in other words, a sample that could be composed both by normal and pathological subjects. In the case of mixed samples, Table 1 reports sizes for both normal and clinical groups.

**Figure 2:**
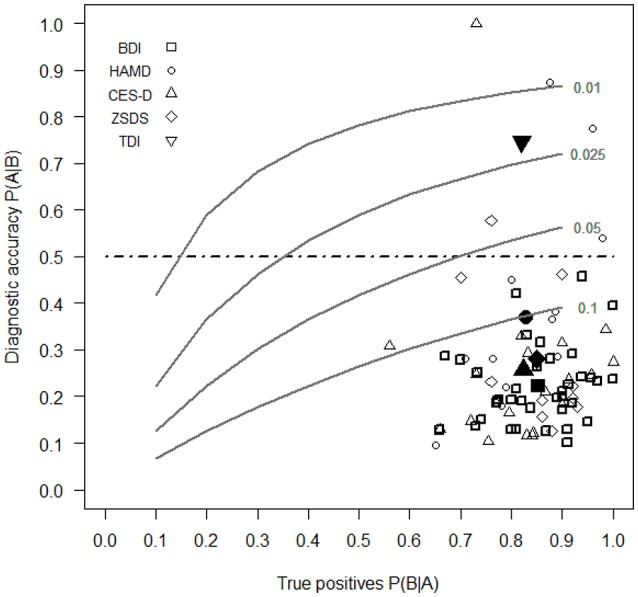
Diagnostic accuracy of BDI, HAMD, ZSDS, CES-D, and TDI estimated with the Bayes' theorem (Equation 1) against the different levels of P(A|B) (horizontal axis) and of FPs (series of gray curves). Open symbols report data for each study, while filled symbols indicate the mean values for each depression scale. For the TDI there is currently one study which reports its sensitivity and specificity values. The selected levels of FPs were 10, 5, 2.5, and 1%.

Figure 2 shows the values of P(A|B) against those of P(B|A) for each selected work of depression scales.

Our results show that the depression scale with the greatest diagnostic accuracy or the highest value of P(A|B) is the TDI. It is the only self-report depression scale that has a percentage probability of diagnostic accuracy of 74.6%, which overcomes the limit of 50% of correct diagnosis according to Bayes' theorem. This means that the true risk of wrong diagnoses with the TDI is 25.6%. In other words, the TDI is the only scale for which if an individual obtains an high score, then he has also a probability to be truly depressed more than that obtained by chance only. The mean percentages of P(A|B) for the BDI, HAMD, ZSDS, CES-D are 22.4, 37.1, 28.0, and 25.7%, respectively, all lower than the value of 50%.

**Figure 3:**
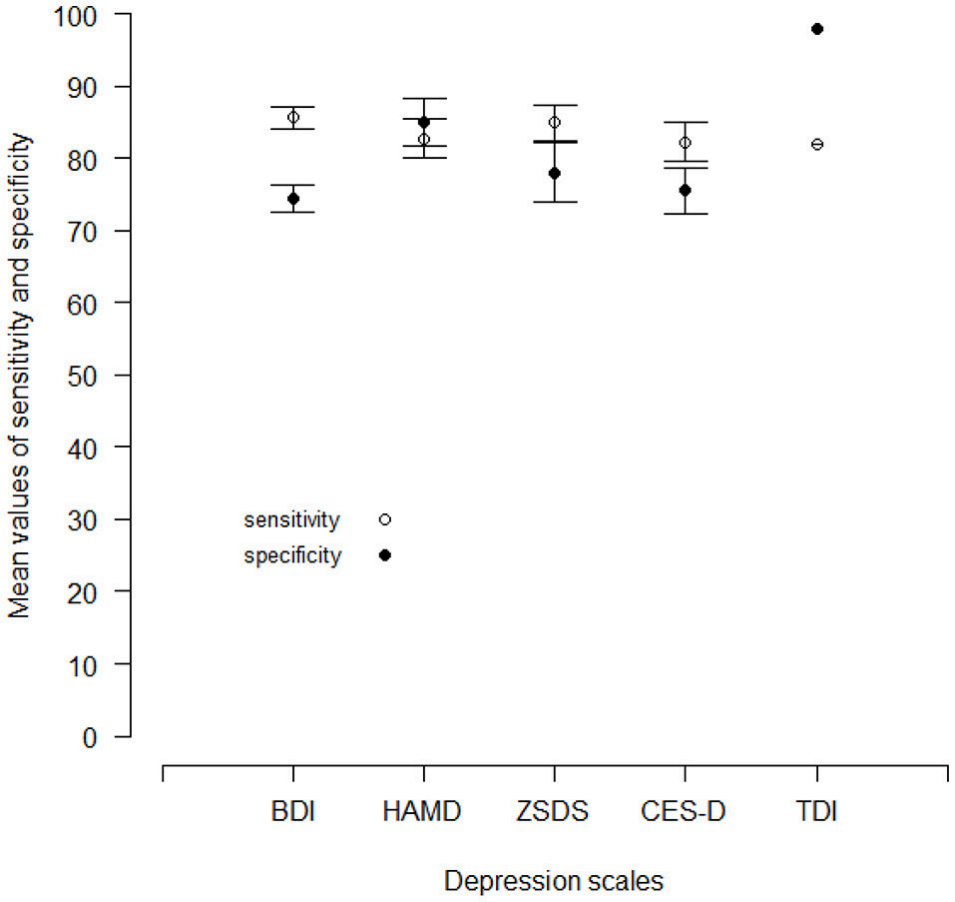
Mean values of sensitivity and specificity for the BDI, the HAMD, the ZSDS and the CES-D reported by different published works. For the TDI, there is currently one published work. Vertical lines are standard errors.

Figure 3 shows the mean values (in percentages) of sensitivity and specificity of each depression scale. All the depression scales have similar values of sensitivity, but two scales, the HAMD and the TDI, have also specificity values greater than the respective sensitivities.

Therefore, only a test with a high level of specificity can guarantee a high level of diagnostic accuracy. In other words, diagnostic reliability can be assured by tests with a small number of FPs.

Table 2 shows the mean values of sensitivity, specificity and cutoff values for the BDI, the HAMD, the ZSDS, the CES-D and the TDI for the three different typology of samples used by authors to estimate test reliability, which are reported in Table 1.

**Table 2:** Mean values of specificity, sensitivity and cutoff values for each kind of sample (normal, psychiatric or medical) used in studies to estimate the criterion validity of depressive scales.

		**BDI**			**HAMD**			**ZSDS**			**CES-D**			**TDI**		
	**Sample typology**	**Sensitivity**	**Specificity**	**Cutoff**	**Sensitivity**	**Specificity**	**Cutoff**	**Sensitivity**	**Specificity**	**Cutoff**	**Sensitivity**	**Specificity**	**Cutoff**	**Sensitivity**	**Specificity**	**Cutoff**
Means	Normal	76.04	81.06	13.60	96.00	98.00	12.00				86.70	76.60	16.00			
	Psychiatric	85.56	70.05	17.35	85.06	89.59	12.33	86.60	77.70	45.00	86.54	69.67	18.07	82.00	98.00	36.00
	Medical	87.25	76.57	13.75	79.03	81.71	10.50	83.22	78.33	51.80	79.23	79.14	16.73			
Standard deviations	Normal	9.57	9.86	4.51	0	0	0				0	0	0			
	Psychiatric	8.35	12.04	6.52	7.78	8.10	2.78	9.48	16.06	4.89	7.43	15.33	7.89	0	0	0
	Medical	8.14	10.31	4.72	7.81	11.06	4.21	7.03	11.28	6.10	13.01	12.44	3.90			
Number of works in which samples are present	Normal	5			1			0			1			0		
	Psychiatric	11			4			5			7			1		
	Medical	20			10			5			11			0		

*Samples cited in only one work have null standard deviation*.

When psychiatric samples are used, depression scales tend to have higher cutoff values, higher sensitivity values and lower specificity values in relation to those of normal and medical samples. Only the HAMD and the TDI have an opposite trend.

## Discussion and conclusion

On the basis of our analyses, all the most famous self-report depression scales, the BDI, the HAMD, the ZSDS and the CES-D have a low diagnostic accuracy, because none of them can arrive to a level of diagnostic accuracy higher than 50%, according to Bayes' theorem. The TDI, which is the last created depression scale, can overcome the 50% level and it is certainly the most reliable test for diagnosis accuracy of depression in comparison with the other scales. In order to overcome psychometric weaknesses of existing measures of depression (Balsamo and Saggino, 2007), TDI was developed by using the IRT procedure (Embretson and Reise, 2013) to select items which best discriminate between normal and clinical patients (Balsamo and Saggino, 2013, 2014). In general, the IRT procedure allows the estimation of items parameters: the difficulty parameter and the discrimination parameter (Embretson and Reise, 2013). The difficulty parameter defines the order of difficulty of test items, while the discrimination parameter allows the selection of items which best discriminate between different groups of subjects (e.g., normal vs. clinical subjects). The IRT procedure used in the development of the TDI can have improved its diagnostic accuracy by reducing the number of FPs. Even if, probably, studies replications are necessary to obtain greater confidence about the diagnostic accuracy of the TDI, however this finding is strongly encouraging about the use of the scale in clinical settings. Therefore it would be preferable when developing new psychological scales of depression to use the IRT procedure in order to select the items which allow the reduction of FPs frequency.

With medical samples the mean values of specificity of depression scales tend to be higher in relation to other samples, except for the HAMD and the TDI. Probably this is due to the fact that medical symptoms are more clearly defined than psychiatric symptoms, allowing a selection of clinical subjects that are representative of the pathological population. By combining these results with the previous mean values of sensitivity and specificity for each scale, it is possible to state that the low diagnostic accuracy of self-report depression scales are due predominantly to the excessive numbers of FPs in samples, especially in those composed by psychiatric subjects, and that this is due, probably, also to an unsatisfactory definition of the fundamental symptoms that best divide the normal subjects from the pathological ones. Thus, it is fundamental to pay attention to the way subjects are selected to compose the clinical samples that are necessary to validate tests which are provided also for use in clinical psychology or psychiatry. Clinical samples, in particular, should be composed by subjects whose symptoms have been clearly defined according to specific criteria as, for example, those specified in the DSM (American Psychiatric Association, 2013) or ICD-10 (World Health Organization, 1992). Otherwise it is possible to include in the clinical sample people that are not representative of the population with that specific pathology, increasing, in this way, the frequency of FPs. In the case of depression scales, the low specificity in psychiatric samples can be due to the fact that some clinical subjects were not truly depressed because the specific symptoms of depression were not accurately determined. Only with a clear definition of pathological symptoms it is possible to have an accurate estimation of the proportion of TPs and FPs. The problem to define the signs to decide correctly the presence or the absence of a medical or a psychological disorder or a malfunction in instruments are well known in literature (Begg, 1987; Hui and Zhou, 1998; Zhou, 1998; Sheppard and Kaufman, 2005). Therefore, psychologists should define clearly the symptoms of psychological diseases to create valid clinical samples that are truly representative of pathological populations. There were some cases in which psychologists obtained high level of diagnostic accuracy when estimating the validity of the depression scale. Figure 2 shows that in some works the depression scales obtained values of P(A|B) higher than 50% (3 cases for the HAMD, one for the ZSDS and one for the CES-D). However in all the remaining cases, diagnostic accuracy still remain unsatisfactory for depression scales (lower than 50%). Probably in these cases, which are the majority, there was not a really accurate selection of clinical subjects.

Our conclusion is that the diagnostic accuracy of a self-report depression scale, but also for every other kind of psychological test with a possible use in diagnostic settings, can be reached on the basis of the bayesian approach. Using Bayes' theorem, it is possible to define the level of diagnostic accuracy of the test and to intervene, in case of low accuracy, on the factors that have reduced its level of accuracy. The most probable factors influencing diagnostic accuracy are the percentage of TPs, or the number of subjects with a real psychological disease that should obtain scores in psychological tests significantly higher than cutoff values, and the percentage of FPs, or the number of subjects without any psychological disease, that run the risk to be incorrectly diagnosed as pathological. The correct determination of the frequencies of TPs and FPs is based on the accurate selection of test items. This accurate selection can be accomplished not only with the best statistical analyses, but also with a clear definition of the symptoms of psychological syndromes. The diagnostic accuracy can be achieved only with the selection of pathological samples which are representative of the pathological population and with the selection of items which can reduce and increase the frequency of FPs and TPs, respectively.

There are other methods to improve diagnostic accuracy in addition to Bayes' theorem (Begg, 1987; Hui and Zhou, 1998; Zhou, 1998), but these methods use complex mathematical procedures and needs specific computer programs which can induce a negative attitude in clinical psychologist toward them, while the Bayesian approach is relatively simple to comprehend and to handle, also by people without great expertise in mathematics and statistics. Our intent was to increase in psychologist who uses self-report tests for their diagnosis the awareness that they should be more confident in tests in which more efficient statistical techniques were used to select items (e.g., using IRT procedures), that they should control if signs or symptoms of psychological syndromes were accurately defined in the clinical sample and that the presence or absence of syndromes in individuals could not be based only on the comparison of individual test scores to the recommended cutoff values.

## Author contributions

MT concept of the work, writing of the work, data analysis. GF writing part of the work, contribution in data analysis. AS proofread of the work, contribution in data analysis.

### Conflict of interest statement

We declare a potential conflict of interests for some authors, those who have published the handbook for one of the tests analyzed in the present report (AS–please see References's section). The remaining authors declare that the research was conducted in the absence of any commercial or financial relationships that could be construed as a potential conflict of interest.
